# Diversity competence in medical education: short-term effectiveness of an interprofessional diversity-specific undergraduate learning

**DOI:** 10.1186/s12909-025-06824-5

**Published:** 2025-02-12

**Authors:** Philipp Linde, Houda Hallal, Polina Charkina, Anne Adams, Julia Frank, Simone Wegen, Jiaqi Fan, Lukas Nadjiri, Heike Zims, Christoph Stosch, Christian Baues

**Affiliations:** 1 Department of Radiation Oncology, Cyberknife and Radiation Therapy, Faculty of Medicine and University Hospital of Cologne, University of Cologne, Kerpener St 62, Cologne, 50937 Germany; 2 University of Cologne, Faculty of Medicine, Office of the Vice Dean for Teaching and Studies, Faculty of Medicine and University Hospital of Cologne, University of Cologne, Kerpener St 62, Cologne, 50937 Germany; 3 Institute of Medical Statistics and Computational Biology, Faculty of Medicine and University of Cologne, Kerpener St 62, Cologne, 50937 Germany

**Keywords:** Medical education, Radiation oncology, Teaching, Medical students, Curriculum, Diversity, NKLM, Medical studies

## Abstract

**Background:**

Diversity competence, diversity itself, and a corresponding awareness of possible (intersectional) discrimination mechanisms have not been anchored in the *German National Competence based Learning Objectives Catalogue for Medicine 2.0 (Nationaler Kompetenzbasierter Lernzielkatalog 2.0., NKLM)* yet*,* highlighting a systemic gap in national competency frameworks. We present our first experience with a prospective diversity-specific intervention in medical students to assess its short-term impact on students' diversity acceptance (DA) and to develop actionable recommendations for integrating diversity into medical education.

**Methods:**

We designed a prospective cohort study using a control group (CG) and intervention group (IG) design. The IG absolved a five-day diversity-specific intervention (50 h; field trip; seminar). Quantitative data were collected using the validated DWD-O5 scale at baseline (T0), three months (T3), and six months (T6), complemented by qualitative responses (diversity issues in the medical curriculum; perceptions and criticisms) categorized using Mayring’s content analysis. Descriptive and non-parametric statistics were performed.

**Results:**

Thirty-one medical students (*n* = 10, IG vs *n* = 21, CG) were enrolled. The IG demonstrated a short-term improvement in diversity competence (+ 9.72%) across all DWD-O5 factors during the intervention. While scores slightly declined at T6, they remained above baseline levels. 35% (CG) vs. 56% (IG) have experienced discrimination in context of medical studies on their own. Participants in both groups stressed the importance of integrating diversity criteria into curricula at an early stage (100% agreement). Findings revealed three key themes: perceived inadequacies in current curricula, self-reported discrimination experiences, and a strong desire for practical diversity training, such as simulation-based learning.

**Conclusion:**

The intervention shows promise as an initial step toward addressing diversity gaps in medical education. By combining historical, cultural, and experiential learning approaches, the program fosters essential competencies such as empathy, self-reflection, and bias recognition. More broadly, sustained improvements in diversity competence require longitudinal integration of diversity training across curricula and systemic reforms to national frameworks like the NKLM. Future research should explore the long-term impact of such interventions and strategies for institutionalizing equity-focused medical education.

## Background

Increasing socio-cultural, economic, gender, and religious diversity underscores the need for equity, diversity, and inclusivity in medical education in Germany [1–6]. Embedding these principles into curricula is essential for preparing future medical professionals [7, 8]. It equips them to address patients’ unique needs, foster inclusivity, reduce health disparities, and improve outcomes [9–11].

**Fig. 1:**
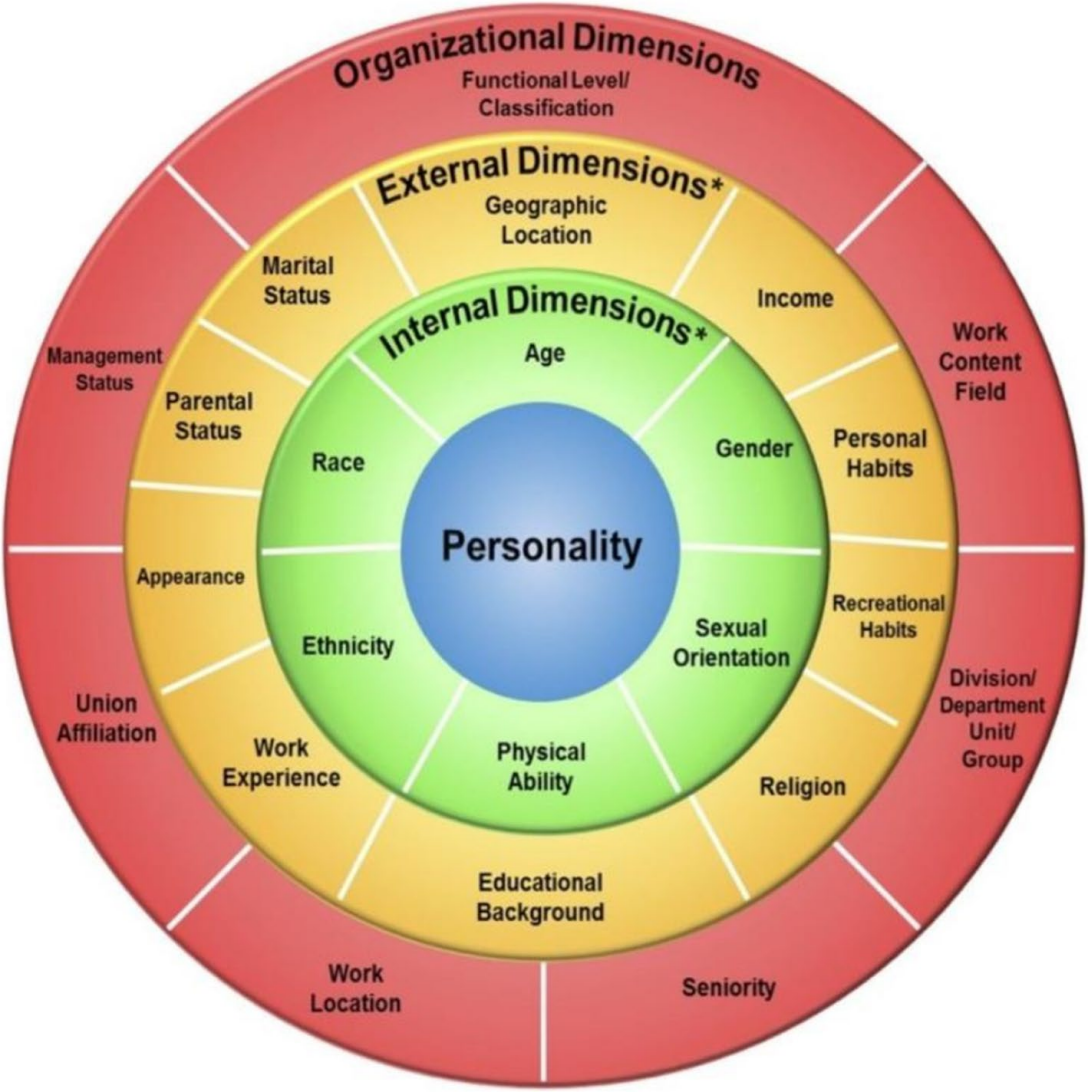
The four-dimensional diversity model of Gardenswartz and Rowe [14–17]

Although patient populations are becoming more diverse, medical education in Germany still lacks strong integration of cultural competence and diversity topics. The “Four Layers of Diversity” model by Gardenswartz and Rowe provides a framework for addressing this gap by examining diversity across interconnected dimensions, from personal traits to organizational roles (see Fig. 1) [12, 13]. This model supports the learning intervention and helps integrate diversity into curricula.

The German National Competence-based Learning Objectives Catalogue for Medicine 2.0 (NKLM) outlines competencies for medical students. In preparation for this study, the author team conducted an independent keyword search for the (German) terms “Diversität,” “diversity,” and “Vielfalt” within the current version of the NKLM. The search yielded limited results, identifying only three general headings and five references to learning objectives in chapter VIII (see Table 1). These findings, as highlighted by Ludwig et al., underscores the urgent need for targeted interventions to better align medical training with the competencies necessary for equitable healthcare [18]. Ongoing revisions to the NKLM must prioritize the systematic integration of diversity across curricula [14, 15, 19].

**Table 1:** Diversity-sensitive (learning) objectives in the NKLM 2.0 (version 03-2022)

**ID**	**Heading**
VIII.2-05.2	Sociocultural diversity
VIII.3	Interprofessional competences
VIII.6	Professional action and ethics, history and law of medicine
**ID**	**Learning objective → The medical students …**
VIII.2-05.2.3	can acquire knowledge about socio-cultural diversity while remaining critical of stereotypes.
VIII.3-01.2	take into account the diversity of the individual members of the team and of the patients and cultivate value-oriented interaction with each other.
VIII.3-01.2.2	are able to recognise socio-cultural characteristics of the person being treated in interprofessional cooperation and to take into account the possible effects on the intended treatment goal.
VIII.6-04.1.2	take into account diversity-related aspects of the patients.
VIII.6-04.4.13	can recognise disadvantages, stigmatisation and discrimination on racist grounds or because of ethnic origin, gender, religion or belief, disability, age or sexual identity and direct action in the sense of preventing or eliminating these disadvantages.

To address these gaps, this study evaluates the *Medicine Meets Art and Culture (Medizin trifft Kunst und Kultur, METRIK* ^*2*^ *)* intervention, designed to enhance diversity competence by integrating historical, cultural, and clinical perspectives into medical education. Using the “Four Layers of Diversity,” this intervention addresses NKLM deficiencies and provides actionable recommendations for embedding diversity into curricula. Specifically, this prospective cohort study investigates the short-term effects of METRIK^2^ on medical students’ acceptance of diversity (DA) while providing actionable strategies for integrating diversity-related topics in medical training. By addressing deficiencies in the NKLM through a structured and evidence-based approach, this study contributes valuable insights to advance diversity-sensitive medical education.

## Methods/project description

### Study design and setting

This research was structured as a prospective cohort study employing both qualitative and quantitative descriptive approaches to measure the impact of a diversity-specific educational intervention. The study was conducted at the Medical Faculty of the University of Cologne, utilizing Kern's six-step approach to curriculum development [20]. This methodological framework was chosen to ensure a systematic design and implementation of the educational intervention, which is aimed at integrating diversity training into medical education. Of note, the primary focus of the study was on evaluating overall diversity acceptance and competence. Diversity-related demographic data, such as ethnicity, were not collected to preserve participant anonymity.

### Intervention description

Medicine Meets Art and Culture (METRIK^**2**^**):** Developed by the Department for Radiation Oncology at the University Hospital of Cologne (PL), this intervention spanned five days and included both seminar sessions and a field trip to Munich, Germany. The content focused on medical practices during the era of National Socialism, the role of radiation oncology in that period, and broader issues of diversity and racism in historical and contemporary contexts [21–25]. The course was designed to foster critical thinking and self-reflection among participants about the ethical dimensions of medical practice. Various interactive elements, such as discussions of historical sources, thematic exhibitions, and theatre performances, were incorporated to enrich the learning experience.

The intervention also sought to inspire participants to explore careers in radiation oncology by emphasizing its historical developments and modern applications as a pillar of tumor treatment. Experts accompanied the field trip, providing interdisciplinary insights and facilitating discussions amongst students [26, 27].

Specific objectives included fostering awareness of diversity-related historical injustices, developing skills to address discrimination in healthcare, and enhancing critical thinking about cultural competence in medical practice. These objectives were closely aligned with the claims of the Medical Faculty Association of the Federal Republic of Germany ((MFT), Medizinischer Fakultätentag der Bundesrepublik Deutschland) and are detailed in Tables 2 and 3 [27–30].

**Table 2:** Learning objectives of the METRIK² course

Category	Learning Objectives
*Active Curriculum Participation*	Enhance attitudes towards engaging actively in curriculum development with a focus on integrating diversity-related topics into medical education.
*Critical Thinking and Educational Insight*	Foster critical thinking about medical education and practices (cultural competence; diversity management). Gain insights into diversity management in both corporate and clinical settings, emphasizing the ethical responsibilities of healthcare professionals.
*Clinical Knowledge and Application*	Understand and analyze medical conditions such as glioblastoma multiforme through interactive case studies and hands-on exercises. Explore how cultural and social diversity impacts the clinical management of complex diseases. To demonstrate the role of radiation oncology in state-of-the-art oncological treatment.
*Historical Context and Analysis*	Develop a comprehensive understanding of the role of medicine during the Nazi era. Acquire skills to critically analyze historical medical practices, sources and their ethical implications on modern medicine, focusing on lessons learned about discrimination and diversity in healthcare.
*Ethical Reflection and Synthesis*	Synthesize knowledge and insights gained from the field trip to reflect on ethical issues in historical and contemporary contexts. Enhance self-reflection on personal biases and develop a deeper understanding of cultural competence in medical practice.

**Table 3:** Structure of the field trip METRIK^2^ (including pre- and post-meeting, Cologne)

	*Modules*	*Learning*	*Teaching methods*
*Preparation meeting*	Experience exchange (possibilities and impact of participation for students in curriculum development)	Attitudes	Small groups
	Keynote lecture working group "Curriculum Development", Critical Medicine^1^, Cologne.	Attitudes	Small groups, discussion, reflection
***Arrival***			
* Day 1*	How to deal with diversity in big companies, Roche Holding, Munich-Penzberg, Germany	Insights, Knowledge	Lecture, discussion
	Radiooncology - the clinical example of Glioblastoma multiforme, Novocure Holding, Optune, Munich, Germany	Insights, Knowledge	Lecture, discussion, case studies, hands-on exercises
	Student lectures	Knowledge	Lecture, quiz, discussion
* Day 2*	The history of *Munich* before, during and after the National Socialist era, *NS Documentation Center, Munich*	Insights, Knowledge, Skills	Lecture, discussion, case studies, hands-on exercises, historical guided city tour with a focus on the influences of the Nazi era
***Departure***			
* Wrap-up meeting*	Recap	Insights, Knowledge, Attitudes	Discussion
	Stage play " Bruder Eichmann^2^, by Heinar Kipphardt"	Insights, Knowledge, Attitudes	Discussion (before and after the visit of the drama)

^*1*^https://krit-med.uni-koeln.de

^*2*^*Adolf Eichmann, the planner and organiser of the Holocaust (1906-1962)*

### Participants and recruitment

Eligibility for the study was restricted to medical students who had reached their third clinical semester (or higher), coinciding with their studies in radiation oncology. An invitation for the field trip and subsequent survey was sent via email to all eligible students. Participation was voluntary and limited to ten students due to COVID-19 restrictions, with a first-come, first-serve policy and a waiting list managed through a move-up procedure. Participants for the control group were randomly selected from the pool of students who met the same academic criteria but did not join the field trip. Recruitment emails were sent to all eligible students, with follow-up reminders to maximize response rates.

### Procedure and data collection

The primary instrument for data collection was a structured online questionnaire, developed collaboratively by the research team, experienced in medical education development and addressing health disparities (CS, HZ, HH, PL). In the development, the four-dimensional diversity model of Gardenswartz and Rowe, as well as the group-related anti-humanity short scales were used [14, 15, 31]. Iterative feedback sessions in this process ensured alignment with the study’s objectives, refined question phrasing for clarity and usability, and planning subsequent steps for curriculum enhancement.

#### The questionnaire

The questionnaire served as the primary tool for data collection, carefully designed to assess a range of attitudes and competencies related to diversity within the medical student population. It combined standardized scales with custom questions tailored to the specific needs of the study.

##### Composition

The questionnaire was structured into three main sections, each targeting distinct dimensions of diversity awareness and interaction:

1. Measurement of Diversity Acceptance: The questionnaire included the Dealing With Diversity In An Organisational Context/5 Factors (DWD-O5) scale by Pietzonka et al., validated for both German and English languages (see Table 4) [32, 33]. The scale was chosen to assess the nuanced perceptions and attitudes towards diversity among medical students. It measures five dimensions of diversity attitudes: Bias, Diversity Beliefs, Dealing with Discrimination, Ethical Dealing with Minorities, and the Affective Diversity Component. Each factor is scored from 1 to 5, with higher scores indicating stronger competence in that dimension. A total score is calculated as a weighted average of the factors, ranging from 1 (low competence) to 5 (high competence).

**Table 4:** Items of the DWD-O5 scale

1) I recognise quickly if someone is discriminated against
2) When I see people being discriminated against, I stand up for them
3) People who are different from me make me feel uncomfortable*
4) I am unbiased toward foreigners
5) I am good at integrating outsiders into our team
6) Our society should pay more attention to the needs of minorities
7) I like to get to know strangers, even if they are different from me
8) I am unbiased toward homosexuals
9) To treat minorities with respect should be even more important for companies (The term "company" refers to the University of Cologne, Faculty of Medicine).
10) Human diversity is a positive resource in the working world
11) I only enjoy working with people who share my values*
12) I am unbiased toward disabled people
13) People, who have a high regard for human diversity, have bigger job benefits
14) A professional dealing with minorities in companies should be more self-evident in future (The term "company" refers to the University of Cologne, Faculty of Medicine).
15) I am unbiased toward people with a different religion
* Items with * are to be reversed during the calculation.

*Additional commentary by the authors of this article:*

Adjustment of individual terms in the questionnaire:

*"Foreigners "* = people with a migration background/ BIPoC (Black, Indigenous and People of Color). BIPoC is a positive, political self-designation of racialised, discriminated persons. The term describes a common horizon of experience shared by people who are not white. This arises, for example, from not being granted privileges. This term does not (primarily) describe skin color

*"Homosexuals"* = LGBTQ*. This is the international abbreviation for lesbian, gay, bisexual, trans and intersex, queer. LGBTQ* people have different experiences of discrimination. However, they are often referred together because all these lifestyles contradict the societal heterosexual norm, according to which there are only two genders

*"Minorities" = socially and historically marginalised groups of people*

Of note: The original questionnaire did not use gender-specific language. Since we could not make any changes for reasons of measurability and validation, we added an explanatory text and clearly marked it within the questionnaire (Table 4).

2) Integration of Diversity Aspects into Teaching (Medical Faculty of the University of Cologne): This section sought insights into how diversity topics are currently integrated into the medical curriculum and the perceived effectiveness of this integration. In this segment, medical students were asked to evaluate their experiences and the visibility of diversity issues in their medical education studies, encouraging them to suggest improvements.
3) General Data of the Participant: This section gathered demographic information and general background data to explore correlations between diversity attitudes with various personal factors such as gender and educational background. This data helped the authors contextualize the responses within broader social and cultural frames, but were not part of further analysis.

The questionnaire was tested for practicability and user orientation by three independent persons before circulation. The form was then given to five other independent persons (three medical students and two medical doctors) to determine the average processing time. Feedback from these phases led to refinements in question phrasing and sequence to optimize understanding and response rates.

The comprehensive design of the questionnaire aimed not only to gather quantitative data on diversity beliefs and practices but also to elicit qualitative feedback on personal experiences and perceptions related to diversity in medical education. The inclusion of open-ended questions allowed respondents to provide personal insights and suggestions, enriching the data set with diverse perspectives on improving diversity education in the medical field.

#### Data collection and follow-up

The field trip, part of the intervention group's (IG) activities, was designed to complement the theoretical knowledge provided in the medical curriculum with practical insights. Prior to the field trip, participants were invited to complete the initial survey (time (T) 0) to establish a baseline before exposure to the intervention. This pre-intervention data collection aimed to minimize bias from the field trip experiences.

The control group (CG, not participating in the field trip) included 21 students to ensure a balanced and meaningful comparison. To accommodate potential dropouts and ensure sufficient data, invitations were extended to 240 students, anticipating a response rate of 5%.

Follow-up surveys were administered to the IG three (T3) and six months (T6) post-intervention to assess the lasting impact of the field trip. The CG was initially scheduled to participate in similar follow-ups, but due to low initial response rates and privacy considerations preventing follow-up contacts – intended to ensure participant anonymity by refraining from collecting or storing personal data that would allow future contact – only baseline data (T0) were collected for this group.

### Data analysis

Quantitative data from the questionnaires were analyzed using IBM SPSS Statistics 28.0 (Microsoft Windows). Descriptive statistics such as mean, median, and interquartile ranges were used to summarize the data. The analysis was further deepened by aligning these results with established medical education frameworks [34–37].

Qualitative responses were categorized and analyzed using a mixed deductive-inductive approach using Mayring’s content analysis to systematically identify and categorize themes [38–41]. The “Four Layers of Diversity” model by Gardenswartz and Rowe provided the theoretical framework for the deductive analysis. Based on this framework and the study’s objectives, four predefined categories were developed: Doctor-Patient Interaction, Social and Cultural Context in Medicine, Self-Reflection and Bias Awareness, and Ethical and Historical Dimensions. These categories provided a structured foundation for analyzing participants’ reflections on diversity competence.

Alongside this deductive approach, an inductive process allowed for the identification of emergent themes directly from the data. Open coding facilitated the discovery of new insights, such as participants’ emotional reflections on historical injustices and the perceived barriers to applying diversity principles in practice. These emergent themes complemented the predefined categories, ensuring a comprehensive understanding of the data.

Responses were systematically coded and grouped into categories based on their alignment with predefined or emergent themes. Units of analysis, such as sentences or meaningful phrases, were carefully examined to maintain consistency in coding. To ensure inter-coder reliability, two researchers (PC, PL) independently coded a subset of responses, and any discrepancies were resolved collaboratively. This iterative refinement process allowed for the categories to evolve in alignment with the dataset, enhancing the robustness of the findings.

The final step of the analysis involved identifying patterns and trends across the categories. These were synthesized to provide insights into how participants perceived and internalized the diversity-focused intervention. This structured and transparent approach ensured a systematic analysis of the qualitative data, capturing both predefined and emergent themes relevant to the study objectives.

Finally, the findings from both quantitative and qualitative analyses were compared with the objectives set forth in the German National Competence-Based Learning Objectives Catalogue for Medicine (NKLM), specifically Chapter VIII "Higher-level competencies." The results were discussed in monthly meetings with the entire research team or more frequently when needed.

#### How to … DWD-O5

The overall score of the DWD-O5 scale is calculated by taking the arithmetic means of the individual factors, weighted by their variance explanations:

- Factor 1 "Bias": Items 15, 8, 12, and 4 assess the degree of prejudice against minorities,
- Factor 2 "Diversity beliefs": Items 9, 10, and 13 evaluate the perceived value of social diversity,
- Factor 3 "Dealing with discrimination": Items 1, 2, and 5 focus on conative aspects that facilitate the assessment of specific actions,
- Factor 4 "Ethical dealing with minorities": Items 6 and 14 involve normative considerations addressing social interactions with minorities,
- Factor 5 "Affective diversity component": Items 3, 7, and 11 capture emotional responses in diverse situations.

Comparisons between pre-intervention and post-intervention responses within the IG and between the CG and IG were made to identify changes and trends (DWD-O5). Two items are included inversely in the calculation to ensure the sincerity of responses. The total score of the DWD-O5 scale (calculated as: DWD-O5 = 0.3 × factor 1 + 0.2 × factor 2 + 0.2 × factor 3 + 0.16 × factor 4 + 0.14 × factor 5) ranges from 0 to 5. Higher scores reflect greater acceptance of diversity and competence in diversity-related practices.

### Ethical considerations

The analysis was conducted in accordance with the guidelines of the Declaration of Helsinki. Informed consent was obtained from all participants via a consent box at the beginning of the survey, ensuring their understanding of the anonymity and confidentiality of their responses. Personal data was stored separately from the questionnaires and explicitly excluded from the analysis. Participants were clearly informed through the questionnaire: ‘All personal data will be treated confidentially, anonymously, and separately from the aforementioned questionnaire results.’ Ethical considerations were rigorously observed, particularly in addressing sensitive topics such as diversity and historical injustices. To address potential emotional distress or re-traumatization, participants were explicitly informed – both in the invitation emails and the participant information and consent form – that they could contact the study coordinator for support or to raise concerns. While no fixed debriefing sessions were scheduled, reflective discussions and group-based exchanges were integral to the METRIK^2^ program, providing participants with opportunities to process their experiences in a supportive setting. The study procedures were reviewed and approved by the Ethics Committee of the Medical Faculty of the University of Cologne as part of an *extended curriculum evaluation* (no reference number; study regulations, §15, 1c) [42].

### Quality control and validation

To ensure the reliability and validity of the survey instruments, pilot testing was conducted with a subset of target participants. Feedback obtained was used to refine the questions for clarity and relevance. Additionally, the robustness of the data collection process was reinforced through secure online platforms to prevent data breaches and ensure the integrity of the data collected (SSL-encrypted web server; JavaScript-based platform "Survey Online" (v2021, enuvo GmbH, Zurich, Switzerland)). Only completed surveys were included in the analysis to ensure data quality [43].

## Results

The recruitment period ran from August 1 to December 31, 2021. For all three surveys, the response rate in the IG was 100.00% compared to 8.75% (T0) in the CG. The time to complete the survey was 25 min in mean (range 15 – 35 min). A total of 31 students (ten (IG) vs 21 (CG)) completed the survey. The participant demographics are summarized in Table 5.

**Table 5:** Participant demographics

Demographic Variable	Category	Intervention Group (IG)	Control Group (CG)
**Gender**	Female	8	8
	Male	2	5
	Not answered	–	8
**Semester of Study**	7th Semester	1	9
	8th Semester	–	2
	9th Semester	–	1
	10th Semester	8	1
	11th Semester	1	–
	Not answered	–	8

### DWD-O5 scale

The DWD-O5 scores indicated an increase in diversity competence in the IG at three months (T3) compared to baseline (T0). For instance, scores for ‘Dealing with Discrimination’ increased from 3.30 at T0 to 3.87 at T3. At six months (T6), scores slightly declined but remained above baseline levels, suggesting partial retention of competence. The mean increasement in diversity competence across all five factors was calculated to be 9.72% during the intervention phase (T0 → T3).

Detailed pre- and post-results for each factor in both groups are shown in Table 6. At T0, the average scores of the CG were slightly higher than those of the IG across all factors. For example, the factor “Bias” scored 4.03 ± 0.05 in the IG compared to 4.10 ± 0.06 in the CG. Similarly, “Diversity Beliefs” scored 4.17 ± 0.12 in the IG versus 4.43 ± 0.14 in the CG, while “Dealing with Discrimination” scored 3.30 ± 0.10 in the IG compared to 3.57 ± 0.12 in the CG.

**Table 6:** Mean DWD-O5-scores (± Standard Deviation (SD)) on "Bias", "Diversity-beliefs", "Dealing with discrimination", "Ethical dealing with minorities" and "Affective diversity component" achieved by the participants of the IG and the CG

Factor	Intervention group (IG)^1^			Control group (CG)^2^
	Time 0	Time 3	Time 6	Time 0
Bias	4.03 ± 0.05	4.20 ± 0.07	4.10 ± 0.06	4.10 ± 0.06
Diversity beliefs	4.17 ± 0.12	4.63 ± 0.20	4.27 ± 0.18	4.43 ± 0.14
Dealing with discrimination	3.30 ± 0.10	3.87 ± 0.23	3.63 ± 0.21	3.57 ± 0.12
Ethical dealing with minorities	4.15 ± 0.15	4.70 ± 0.26	4.20 ± 0.22	4.50 ± 0.17
Affective diversity component	3.53 ± 0.08	3.63 ± 0.05	3.60 ± 0.04	3.65 ± 0.09
**Total score DWD-O5 ** **± SD**	**3.85 ** **± 0.11**	**4.07 ** **± 0.18**	**3.98 ** **± 0.16**	**4.05 ** **± 0.12**

^1^IG = Students who participated in the field trip METRIK²

^2^CG = Students who did not participate in the course METRIK² and were randomly selected for the survey

During the intervention, the scores of the IG increased across all measured factors by Time 3 (T3). The largest percentage increases between T0 and T3 were observed for “Dealing with Discrimination” (17.27%, T3: 3.87 ± 0.23), followed by “Ethical Dealing with Minorities” (13.25%, T3: 4.70 ± 0.26) and “Diversity Beliefs” (11.03%, T3: 4.63 ± 0.20). Smaller increases were noted for “Bias” (4.22%, T3: 4.20 ± 0.07) and the “Affective Diversity Component” (2.83%, T3: 3.63 ± 0.05).

Six months after the intervention (T6), the scores in the IG decreased compared to T3. However, all factors retained higher values at T6 compared to the initial scores at T0. For example, the factor “Bias” scored 4.10 ± 0.06 at T6, while “Dealing with Discrimination” scored 3.63 ± 0.21, both remaining above their T0 levels.

**Fig. 2:**
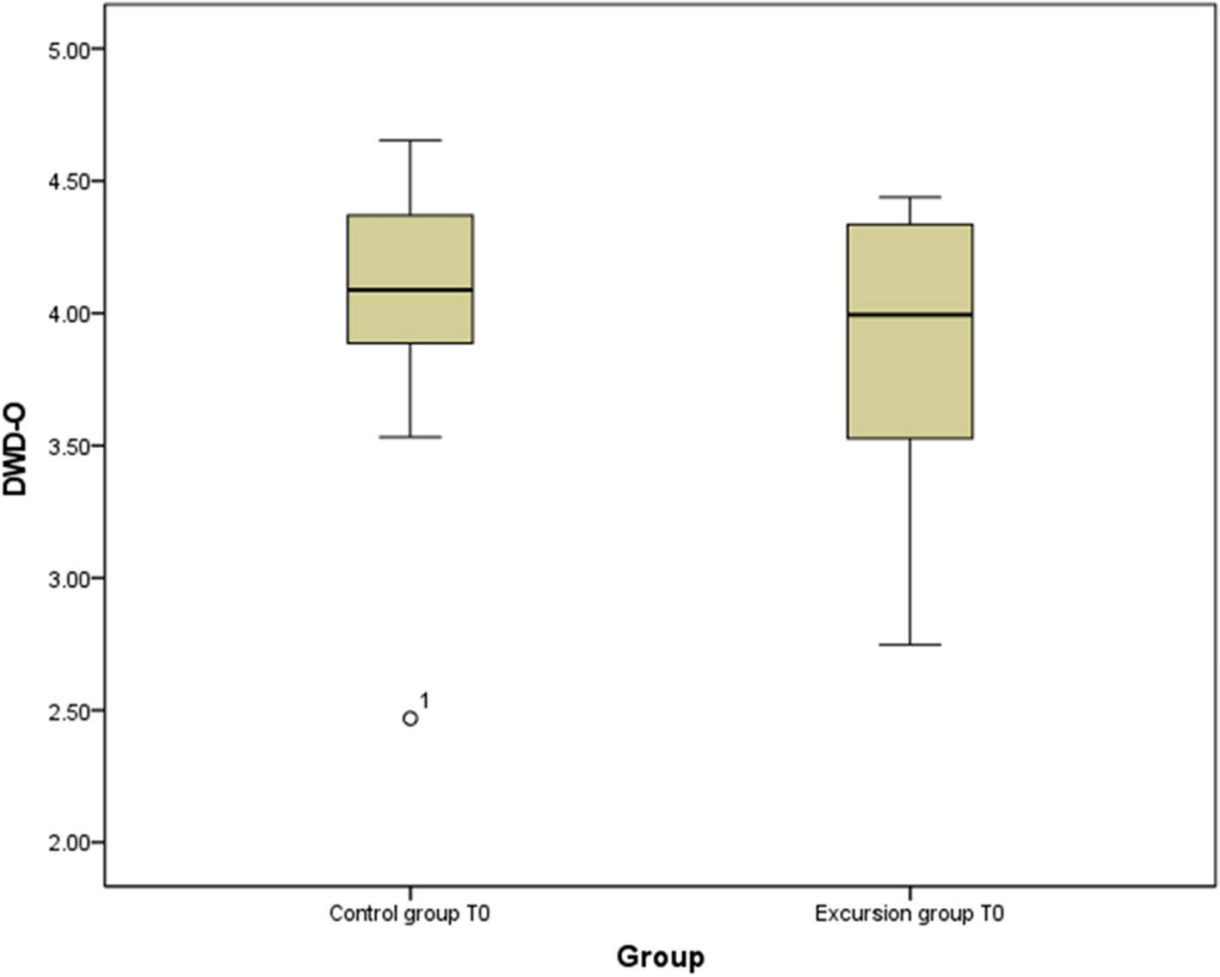
Data distribution in the IG and the CG at the time point T0 (total score)

Figure 2 illustrates the distribution of the DWD-O5 scale data at T0. The median score in the CG is 4.09, compared to 3.99 in the IG. The scoring in the CG is concentrated with an interquartile range of 0.5, compared to 0.75 in the IG. Greater dispersion is observed in the IG, with scores ranging from 2.75 to 4.4. compared to 2.5 to 4.7 in the CG.

**Fig. 3:**
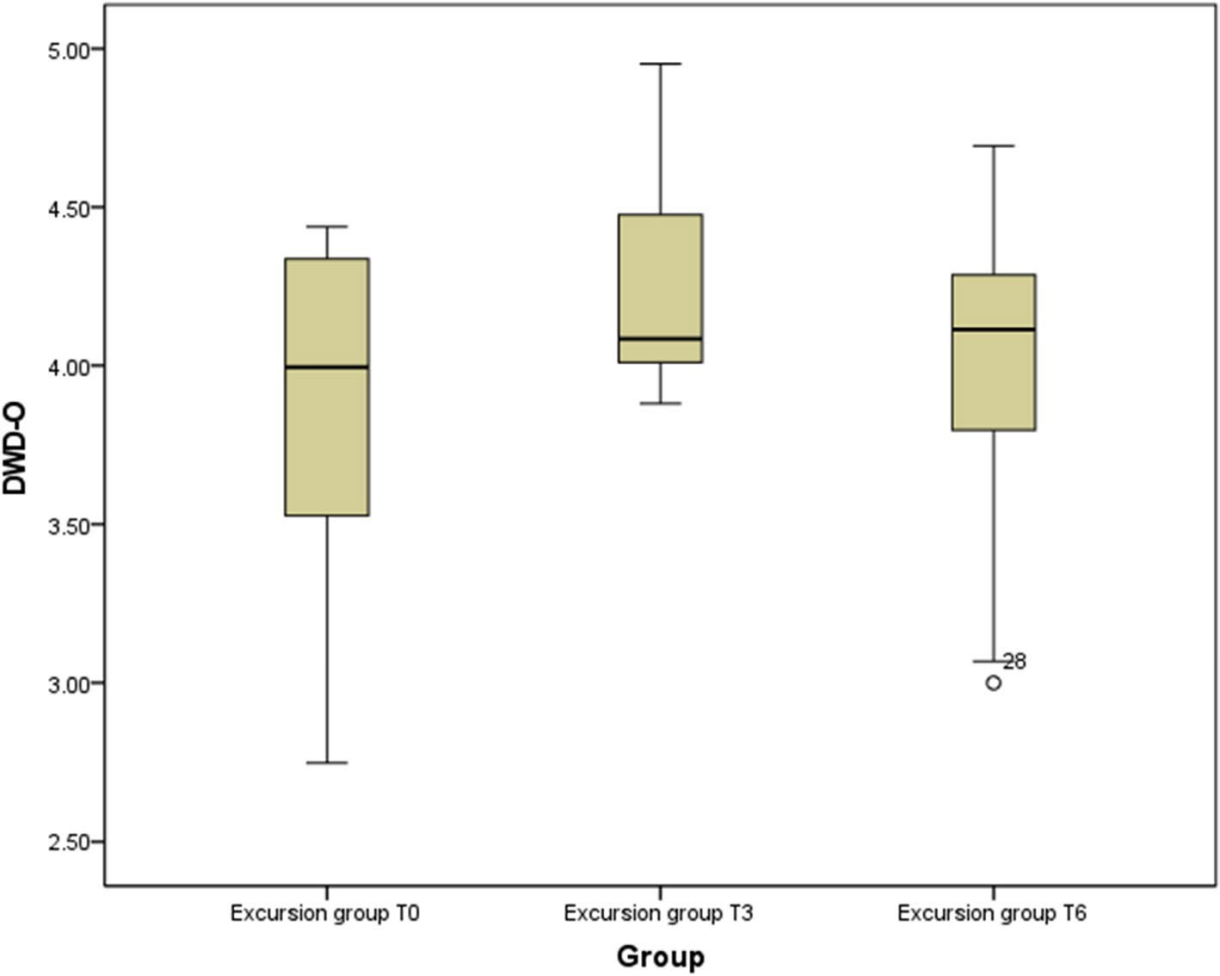
Progress display in the IG at time points T0, T3 and T6 (total score)

Figure 3 demonstrates the median value of the CG at T0. At the three-month follow-up, the median score in the CG increases to 4.25, before declining to 4.10 at T6. The data distribution remains asymmetrical across all time points.

### Exploratory-qualitative analysis

#### Method-related aspects

In the free-text statements on the integration of diversity dimensions in teaching and the longitudinal curriculum at the Faculty of Medicine of the University of Cologne, the participants of both groups emphasized the importance and necessity of integrating diversity criteria into their medical education at an early stage. The students indicated that aspects of diversity are *taught inadequately* or *not all*. They described their current curricula regarding diversity as *superficial, providing just a few practical skills*. It was mentioned that some *feel badly prepared for caring for diverse patients and the different aspects that need to be taken into account*. The students wanted specific training in developing skills in dealing with diversity in the context of their medical studies.

When asked about their needs in patient-oriented diversity within medical education, students emphasized a desire for more practice in simulated settings. This would allow *to practise skills though in an artificial, however life-like diverse situation besides theorising the learned standard*. Simulated scenarios were seen as an important complement to traditional teaching approaches.

Overall, students wished for more communication training. The importance of understanding diversity criteria and incorporating them into medical training as well as future professional practice is highly valued: both groups agreed 100%. In particular, the desire to acquire emotional and social intelligence and to strengthen existing skills was expressed.

#### Content-related aspects

Self-reported reasons for participating in the field trip with its specific topic were mostly interest in the topic itself (90%) and the attractive field trip’s destination (90%). Other reasons for attending the course were the expected relevance for the final exams (radiation oncology, 50%) and the (reputation of the) lecturer (50%). Overall, 85% of the IG indicated that they were already interested in diversity and its core dimensions before participating in the survey (67% in the CG). In addition, 77% of the IG reported having dealt with diversity topics more in detail compared to 57% in the CG.

### Self-revelation of discrimination

35% of participants in the control group and 56% in the excursion group reported experiences of discrimination based on their gender by instructors and colleagues. For example, one female participant was told that women should choose a less demanding specialisation as a doctor because *this would be more compatible with future family planning*. A male student reported how he was asked to undress by the lecturer in an examination course *because his body would please the female students*. One participant reported racist discrimination: colleagues were surprised about his *good language skills despite the dark colour of his skin*. Participants reported that female students were given preferential treatment in practical examinations if they conformed to stereotypical notions in the eyes of the lecturer.

### Required competencies from the student's perspective

When asked to name the competencies and knowledge to understand different cultures and perspectives, four topics could be extracted according to Mayring: 1) doctor-patient communication, 2) medical expertise in the context of social diversity, 3) ability to empathise and change perspective, and 4) ability to self-reflect. The topics could be summarised in (1) the ability to access subject content in the context of social diversity and to transfer it to professional contexts, 2) social and emotional intelligence, and 3) the ability to empathise and adopt a perspective.

## Discussion

Our findings suggest that diversity-specific courses have the potential to enhance medical students’ diversity competence in working with both (future) colleagues and patients. However, given the small sample size and limited scope, these results should be interpreted cautiously. While the intervention demonstrated short-term improvements in DA and related competencies, the sustainability of these changes remains uncertain and requires further longitudinal research.

Our approach in the METRIK^2^ intervention resonates with prior studies, which emphasize the necessity of systematic diversity education in bridging the gap between theoretical knowledge and practical application [44]. Diversity-specific courses are often offered as part of cultural-sensitive courses at universities [45–47]. In line with our data, Crosson et al. showed evidence that medical students’ attitudes, diversity-related knowledge and skills can be positively influenced when participating in cultural-competency courses [48]. Crandall et al. clearly demonstrate positive changes among students in the medical school's approach when integrating cultural competence training [45]. Their study found that students who participated in the program reported more positive changes in their approach to cultural competence than those who did not and felt increased comfort in working with diverse populations. It underscores the importance of embracing cultural and organizational adaptation to foster equitable healthcare practices [49].

Students in our study expressed the value of experiential learning methods, such as simulation-based training (SBT). Participants highlighted the importance of simulations for improving empathy and communication, which may align with the observed increase in DWD-O5 scores for ‘Affective Diversity’ and ‘Diversity Beliefs’ in the IG. This finding aligns with evidence from literature, where simulations and perspective-taking exercises have proven effective in promoting empathy and reducing prejudice [50]. Here, Crisp and Turner demonstrated the effectiveness of perspective-taking in reducing prejudice and discrimination [51]. Their SBT found that perspective-taking increased intergroup cooperation, fostered positive relationships, and reduced prejudice among participants in various settings.

An important finding was our students’ self-reported experiences of discrimination during their own medical training. The most common forms of discrimination were gender-based bias and stereotyping, though racial prejudice and microaggressions were also noted. These observations are consistent with data from other German institutions, such as Charité Berlin and Hannover Medical School, where students have reported similar experiences of inequity and bias [52, 53]. By interviewing more than 30.000 medical students, Weiss et al. demonstrated that racial/ethnic minorities, females and LGBTIQ*-identifying students were more likely to report perceiving a lack of respect for diversity [54]. These findings underscore the critical need for institutional reforms to address systemic discrimination and foster inclusive educational environments [55].

This study identified core competencies regarded as essential for addressing diversity in clinical practice, including effective doctor-patient communication, empathy, self-reflection, and medical expertise in diverse contexts. These competencies are foundational to providing equitable healthcare and should be emphasized across longitudinal medical curricula [56]. Consistent with earlier research, such as Geller et al., our findings support the notion that DA evolves over time, influenced by the learning environment and baseline attitudes at matriculation [57]. Therefore, diversity-specific content must be revisited and reinforced throughout medical training to achieve meaningful change [58].

Although the short-term improvements observed in this study are encouraging, they highlight the limitations of one-time interventions in creating sustained changes in attitudes or behaviors. A more comprehensive, multi-modal approach, incorporating simulation-based exercises, unconscious bias training (UBT), and role modeling, is likely necessary to effect lasting transformation.Role models in medical education with diversity-related knowledge and skills inspire medical students to develop cultural competence, empathy, and adaptability, offering real-life examples of inclusive, high-quality care while addressing challenges in a diverse healthcare environment [59–61]. UBT has demonstrated efficacy in raising awareness of implicit biases and fostering more equitable decision-making in clinical settings [62–65].

The integration of diversity-specific courses into smaller specialties, such as radiation oncology, offers a unique opportunity to embed these principles in areas that are traditionally less diverse in scope. However, responsibility for diversity education must also extend to larger disciplines like internal medicine and surgery, which are integral to broader curriculum development. Smaller specialties can serve as complementary sites for innovation and targeted interventions, thereby alleviating some of the content burden on major disciplines [23, 66]. Incorporating longitudinal diversity training –starting with foundational empathy-building exercises in year one and advancing to cultural competence simulations in clinical years –may enhance systemic integration.

While this study contributes valuable insights, it also raises critical questions about the implementation of diversity education. Our pre-analysis of the NKLM revealed limited integration of diversity-related objectives, with only three general headings and five specific learning objectives identified. The current and common nomenclature in terms of diversity is not used within the NKLM 2.0. This lack of representation highlights the need for systematic integration of diversity topics already in university education, as exemplified by the METRIK^2^ intervention. For instance, courses could include modules on recognizing institutional racism and discrimination. Frameworks such as `critical race theory` and `Four Layers of Diversity` offer practical guidance for confronting biases and embedding equity-focused approaches in medical education and should therefore be incorporated [60]. Raising awareness of injustice and discrimination within our students’ own ranks or towards fellow students will translate into greater sensitivity to these issues in daily patient care and our future healthcare system.

### Strengths and limitations

This study has several limitations that provide valuable insights for refining future research and educational interventions. One notable limitation is the small sample size (*n* = 10), which may affect the generalizability of the findings and its statistical power to perform in-depth quantitative analyses. The study’s reliance on voluntary participation may also have introduced selection bias, as participants with a preexisting interest in diversity topics may have been more likely to engage. Addressing these challenges in future studies by expanding participant cohorts and employing randomized selection methods will enhance the robustness and representativeness of the results.

Privacy considerations posed constraints on follow-up data collection, particularly within the CG, and this impacted the longitudinal assessment of intervention outcomes. To improve follow-up response rates while maintaining anonymity, future research will explore more innovative methods for participant tracking and engagement, such as anonymous identifiers or alternative data collection strategies.

Future studies could incorporate more comprehensive qualitative methods, such as in-depth interviews or focus groups, to capture a broader range of participant experiences and perceptions. Lastly, the study did not include demographic data in its analysis to ensure participant anonymity. While this approach upheld ethical and institutional considerations, it limited the ability to explore diversity through the lens of intersectionality. Future research should consider ethically appropriate ways to incorporate anonymized demographic data, allowing for a more nuanced examination of how different identity factors influence diversity competence.

### Implications for curricular development and research

This study underscores the importance of integrating diversity education into medical curricula to address existing gaps and to promote inclusive healthcare practices. Experiential learning methods, such as SBT and UBT, offer practical strategies for improving students’ competence in handling diversity. However, the findings suggest that a one-time intervention is insufficient for fostering sustained change. Longitudinal, multi-modal approaches supported by institutional policies and equity-focused frameworks are necessary to ensure meaningful and lasting impact.

The findings also highlight systemic inequities in medical education, evidenced by students’ reports of discrimination. Institutions must take active measures to create safe and inclusive learning environments, offering students resources to report and navigate discriminatory experiences. To address systemic discrimination, institutions should implement mandatory anti-discrimination training for both faculty and students. Safe reporting mechanisms for incidents of discrimination should be established, ensuring anonymity and follow-up support. Furthermore, institutions should regularly audit their curricula, teaching methods, and assessment tools to identify and eliminate biases. For example, integrating diversity-sensitive language in exams and clinical case studies can help normalize inclusivity across educational practices. Additionally, national competency frameworks, such as the German NKLM, should prioritize diversity objectives to ensure these topics are adequately represented across medical curricula [67, 68]. The optional catalogues currently available, e.g. LOOOP (Learning Opportunities, Objectives and Outcomes Platform) are a useful resource but insufficient for comprehensive implementation [69].

Aligning these frameworks with best practices, including clear and actionable diversity goals, will help establish diversity competence as a core component of medical education.

## Conclusion

This study provides preliminary evidence of the potential benefits of diversity-specific courses in medical education, exemplified by the METRIK^2^ intervention. While short-term improvements in diversity acceptance were observed, the study’s limitations highlight the need for longitudinal, multi-modal approaches to foster sustained change. By integrating diversity topics into national competency frameworks as the German NKLM, adopting experiential learning methods, and addressing systemic inequities, medical schools can better prepare students to deliver equitable and inclusive patient care. Future research should focus on evaluating the long-term impact of diversity education and developing strategies for its broader implementation.

## Data Availability

Supporting documents are available upon request to the corresponding author.

## Abbreviations

DA: Diversity acceptance
METRIK^2^: Medicine Meets Art and Culture; Medizin trifft Kunst und Kultur
MFT: Medical Faculty Association of the Federal Republic of Germany; Medizinischer Fakultätentag der Bundesrepublik Deutschland
NKLM: National Competence-Based Learning Objectives Catalogue for Medicine 2.0; Nationaler Kompetenzbasierter Lernzielkatalog Medizin 2.0
RO: Radiation oncology
